# Optimal designs of mollusk shells from bivalves to snails

**DOI:** 10.1038/srep42445

**Published:** 2017-02-10

**Authors:** Takuya Okabe, Jin Yoshimura

**Affiliations:** 1 Graduate School of Integrated Science and Technology, Shizuoka University, Hamamatsu 432-8561, Japan; 2 Graduate School of Science and Technology, Shizuoka University, Hamamatsu 432-8561, Japan; 3 Marine Biosystems Research Center, Chiba University, Uchiura, Kamogawa, Chiba 299-5502, Japan; 4 Department of Environmental and Forest Biology, State University of New York College of Environmental Science and Forestry, Syracuse, NY13210 USA

## Abstract

Bivalve, ammonite and snail shells are described by a small number of geometrical parameters. Raup noted that the vast majority of theoretically possible shell forms do not occur in nature. The constraint factors that regulate the biased distribution of natural form have long since been an open problem in evolution. The problem of whether natural shell form is a result of optimization remains unsolved despite previous attempts. Here we solve this problem by considering the scaling exponent of shell thickness as a morphological parameter. The scaling exponent has a drastic effect on the optimal design of shell shapes. The observed characteristic shapes of natural shells are explained in a unified manner as a result of optimal utilization of shell material resources, while isometric growth in thickness leads to impossibly tight coiling.

Shell morphology and its conceptual implications have attracted the attention of scientists in a wide range of disciplines^1^,^2^,^3^,^4^. Computational investigations have aimed at providing realistic descriptions of shapes and patterns of coiled shells^5^,^6^,^7^,^8^,^9^, while empirical investigations have concentrated on the analysis of the adaptive nature of various morphologies^10^,^11^,^12^,^13^,^14^. Although the degree to which evolution is predictable is under debate, the phenomenon of convergent evolution demonstrates that evolutionary pathways are more or less constrained above the level of species^15^,^16^,^17^. In effect, there is evidence that shell shapes are adaptive, for they have evolved independently more than once^18^. Convergent evolution of form is addressed from a modeling perspective of theoretical morphology^12^, in which the concept of theoretical morphospace is introduced^10^. Each point in a morphospace represents a hypothetical form and the evolution of an actual form is visualized as a predictable process in the morphospace^12^,^13^.

The seminal work of Raup showed that natural shells are not randomly distributed in the morphospace of theoretically possible forms, but rather they are confined to restricted regions^10^. The biased distribution is explained in terms of functional and developmental factors in the manner that theoretically possible but naturally not occurring forms would be biologically impossible or functionally inefficient^13^. By investigating various functional factors, Raup concluded that the observed distribution of normal ammonoids, an extinct group of marine mollusks (cephalopods), is not explained by a single factor. Indeed, the ideal form to optimize the utilization of shell material resources did not correspond to any natural species^19^.

The concern of the present study is that this accepted opinion is based on a plausible and convenient assumption of isometric growth that shell thickness increases in proportion to shell size. Although this assumption is often made for spiral shells in general^20^,^21^, as remarked by Raup^19^, biometric data of Trueman indicate rather that shell thickness of ammonoids does not increase as rapidly as shell size^22^. If this observation is accepted, it is not intuitively clear whether and how allometric variation of thickness influences the economy of curved surface construction. Fortunately, this problem is reduced to a well-defined mathematical problem. Here we revisit how shell shape affects shell use efficiency, particularly concerning the effect of allometric scaling of shell thickness. The neglected factor of thickness variation is shown to have a significant impact on the evolutionary perspective of shell form.

Shell form is represented by the growth trajectory of the mouth aperture^23^. We investigate hypothetical shells generated from a circular aperture with radius 1 and thickness *h*_0_ (Fig. 1a). Each form is specified by the center coordinate (*x*_0_, *y*_0_) of this initial aperture, the whorl expansion rate (*W*), and the scaling exponent of thickness (*ε*). The parameter *W* is used by Raup, while the first two parameters *x*_0_ and *y*_0_ correspond to Raup’s *T* and *D* by *T* = *x*_0_/*y*_0_ and *D* = (*y*_0_ − 1)/(*y*_0 _+ 1) (ref. 10). Various shapes are represented by means of these three parameters (*x*_0_, *y*_0_, *W*) (Fig. 1b). The last parameter (*ε*) for thickness variation is a new feature of this study. When the scaling is isometric (*ε* = 1), thickness and size of the aperture grow at the same rate. Allometric scaling (*ε* < 1) means that shell thickness does not increase as rapidly as the apertural size. For a given volume of shell material (*V*_*s*_), different shells with different sets of “genetic” instructions (x_0_, y_0_, *W* and *ε*) end up with different interior volumes (*V*). The present problem is to find optimal shape to maximize the inner volume (*V*) for a fixed volume of shell material (*V*_*s*_). A scaling argument indicates that these volumes *V* and *V*_*s*_ are proportional to the 3rd and (2 + *ε*)-th power of linear size *L*, respectively. Roughly speaking, the latter is understood as surface area (*S *∝ *L*^2^) times thickness (*h *∝ *L*^*ε*^), i.e., *V*_*s*_* *∝ *L*^2+*ε*^. We are interested in shape dependence, i.e., the problem independent of the size *L*. Maximizing inner volume (*V*) for a given value of shell volume (*V*_*s*_) is equivalent to maximizing 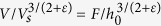, where a factor *F* is introduced by noting that *V*_*s*_ is proportional to *h*_0_, the initial thickness (Fig. 1a). This factor is determined by the shell form (*x*_0_, *y*_0_, *W*, and *ε*) (Supplementary Information). Accordingly, it is interpreted as a measure of the efficient use of shell materials. Below we show how this factor (*F*) varies depending on the morphological parameters (*x*_0_, *y*_0_, *W*, and *ε*).

Figure 2 shows contour plots of *F* in the *x*_0_-*y*_0_ plane for various values of *ε* and *W*, where the expansion rate *W* is expressed in terms of the natural logarithm log*W*. Three bottom panels for isometric growth (*ε* = 1) indicate that the peak of *F* does not lie in the shown range of *W*. Indeed, *F* is maximized for log*W* = 0 (*W* = 1). Shell shapes in this limit are unrealistically tightly coiled. Most importantly, a sublinear variation of thickness (*ε* < 1) brings an optimal shape in a realistic region of the parameter space (morphospace). For *ε* = 0.5 (the second row of Fig. 2), a peak of *F* lies at (*x*_0_, *y*_0_, log*W*) = (0, 1, 2.83).

Optimal values of the morphological parameters (*x*_0_, *y*_0_, *W*) vary depending on the thickness exponent *ε* in an interesting manner. As shown in Fig. 3a, optimal shape makes transition around 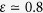 from a plane-spiral form (*x*_0_ = 0) to a conical-spiral form (*x*_0_ > 0). For *ε* < 0.6, *F* is maximized at *x*_0_ = 0 and *y*_0_ = 1 (Fig. 3b). Then the expansion center *O* lies at the edge of the aperture (*D* = 0). This is a basic characteristic of bivalve shell form (*ε* = 0 of Fig. 3a)^1^. As *ε* increases, a steep rise in *y*_0_ sets in while keeping *x*_0_ = 0 for a while. A leftward rise in Fig. 3b is continued to a dashed line in Fig. 3c. Note that *W* on the horizontal axes of Fig. 3b and c decreases as *ε* increases. Figure 3d is a contour plot of *F* in the *W*- *y*_0_ plane for *x*_0_ = 0, where an adaptive ridge of plane spiral (planispiral) forms is seen. As a matter of fact, for *ε* > 0.8, the planispiral form is barely stable if it were not for the constraint of bilateral symmetry (*x*_0_ = 0). The true optimum has a conispiral form (*ε* = 0.9 in Fig. 3a). Two ridges radiate from the summit of *F*, one circling to the planispiral *x*_0_ = 0 and the other flowing along the *x*_0_-axis (see panels for log*W* = 0.55 in Fig. 2). As *ε* increases further, the optimum shifts in the latter direction. Figure 3e shows the *W*-dependence of the semi-angle *β* at the cone apex of optimal shape. In the limit of isometric growth (*ε *→ 1), optimal shape is infinitely highly spired, i.e., *W *→ 1, *x*_0_* *→ ∞, *β *→ 0, and *F *→ 0.85. This is consistent with the above observation that the ideal isometry gives an unreasonable result. In effect, this limit is a mathematical singularity (Supplementary Information). Even a slight deviation from isometry (*ε* = 1) has a significant effect on optimal shape. Figure 3f shows the locus of optimal points in the morphospace.

Even though there is no adjustable parameter, the results conform with general tendencies observed in the frequency distribution of actual species. In Fig. 3c and e, biological data are indicated for comparison^1^,^19^. Raup remarked that the bulk of species in four taxonomic groups, brachiopods, bivalves, gastropods, and cephalopods, are confined to non-overlapping regions, which if taken together comprise a relatively small part of the parameter space^10^. These regions are indicated in Fig. 3f. The result in Fig. 3c is consistent with the prior result *W *→ 1 and *D *→ 1 for isometric ammonoids (*ε* = 1)^19^. For conical spiral shells, the result in Fig. 3e accords with the tendency that high-spired gastropod shells are constrained to have a low expansion ratio (negative correlation between *T* and *W*)^24^. In the continuous spectrum of our solutions, the basic forms of snails and bivalves lie at the opposite ends (Fig. 3). In prior studies, it was necessary to fix *W* at a realistic value^20^,^25^. Assuming 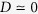 from the empirical facts, McGhee showed that fossil biconvex brachiopods tend to optimize the surface-to-volume ratio^12^,^13^. In contrast to these studies, optimal values of *W* and *D* are uniquely determined without ad hoc assumptions (Fig. 3f).

In the present model, the shell volume increases as *V*_*s *_∝ *L*^2 + *ε*^ in terms of a linear size *L*, which may be of use to evaluate the thickness exponent *ε* empirically. The exponent appears to have not been investigated except for ammonoids^19^,^22^. As remarked by Raup^19^, their thickness exponent *ε* is certainly less than 1 and most likely *ε* ≅ 0.8 (Supplementary Fig. S2). Indeed, Trueman^22^ observed that the volume of ammonoid shells increases in proportion to the diameter raised to the power of 2.7–2.8. In comparing with actual shells, however, the thickness variation *h* ∝ *L*^*ε*^ (Fig. 1) does not necessarily correspond to the size dependence of actual thickness if the animal overlays or redistributes shell material as it grows. In fact, thickness of a clam shell is made approximately constant by accretion of an inner shell layer, whereas total thickness increases as size increases^26^. This observation is consistent with 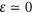, while the constant thickness *h*_0_ in this case is actually not constant for shells of different sizes. Biology is often regarded as a science of exceptions, defying a unified theory. As a pure theory of a general nature, the present study is primitive in many other regards. Additional parameters are necessary to describe more detailed aspects of real forms^8^. At a low phylogenetic level, ontogenetic variation in the parameters may not be negligible^19^,^27^. The efficiency of shell material use is only one factor in the evolution of coiled organisms. In fact, diverse forms in nature signify the concomitant presence of competitive driving forces and various constraints. To name a few, a small value of *W* is unfit for bivalves to hold the two valves together^12^,^13^. Hydrodynamic efficiency should contribute to ammonoid forms (Fig. 3c and d)^11^,^12^,^13^. Postural stability may compete with the shell use efficiency depending on the mode of life (Fig. 3e)^14^. The actual optimality of shell forms can thus depend on various life history traits and environmental factors, such as predatory animals, food habits and habitat conditions.

It has long been considered that (i) isometric growth is a sound assumption in coiled shells and (ii) shell shape has to do with optimal utilization of shell material^19^,^20^,^25^. We showed that these two conceptions are compatible only if non-isometric thickness variation is taken into account. The more the ideal isometry is approached, the more curved the optimal shell surface.

## Additional Information

**How to cite this article:** Okabe, T. and Yoshimura, J. Optimal designs of mollusk shells from bivalves to snails. *Sci. Rep.* **7**, 42445; doi: 10.1038/srep42445 (2017).

**Publisher's note:** Springer Nature remains neutral with regard to jurisdictional claims in published maps and institutional affiliations.

## Supplementary Material

Supplementary Information

## Figures and Tables

**Figure 1:**
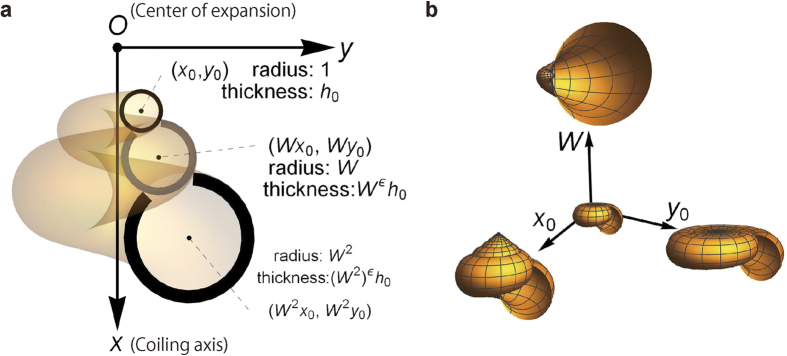
Theoretical representation of coiled shells. (**a**) A coiled shell is described by geometrical parameters *x*_0_, *y*_0_, *W, h*_0_ and *ε.* The first two parameters (*x*_0_, *y*_0_) are the *x* and *y*-coordinates of the center of an initial aperture of radius 1 and thickness *h*_0_. The expansion rate of successive whorls is *W,* whereas thickness varies in proportion to *W* raised to the power of *ε.* As shown in this figure, successive whorls overlap when the expansion rate *W* (>1) is small. (**b**) Coiled shell forms in the three-dimensional parameter space (morphospace) of *x*_0_ (>0)*, y*_0_ (>0) and *W* (>1). This is a schematic representation of varying morphology. The central form is continuously deformed into each of three forms at the end of axes as one of the three parameters *x*_0_,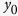 and *W* is increased while the others are fixed.

**Figure 2:**
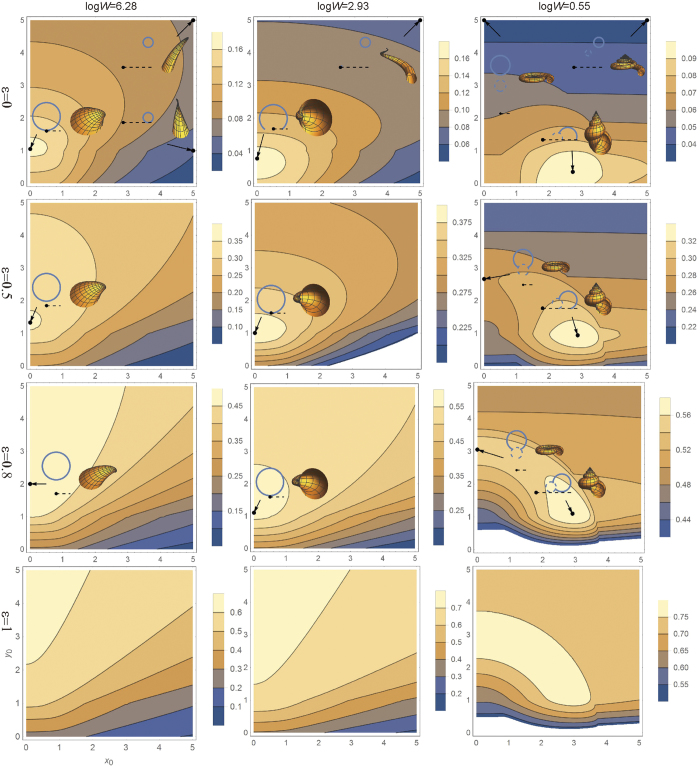
Shape dependence of shell material efficiency. The efficiency (*F*) is shown as contour plots in the *x*_0_-*y*_0_ plane for *ε* = 0, 0.5, 0.8, 1 and log*W* = 6.28, 2.93, 0.55. For some representative points (indicated with arrows), the aperture (circle) and shell shape (image) are shown along with the coiling axis and the expansion center (a dot with a dashed line).

**Figure 3:**
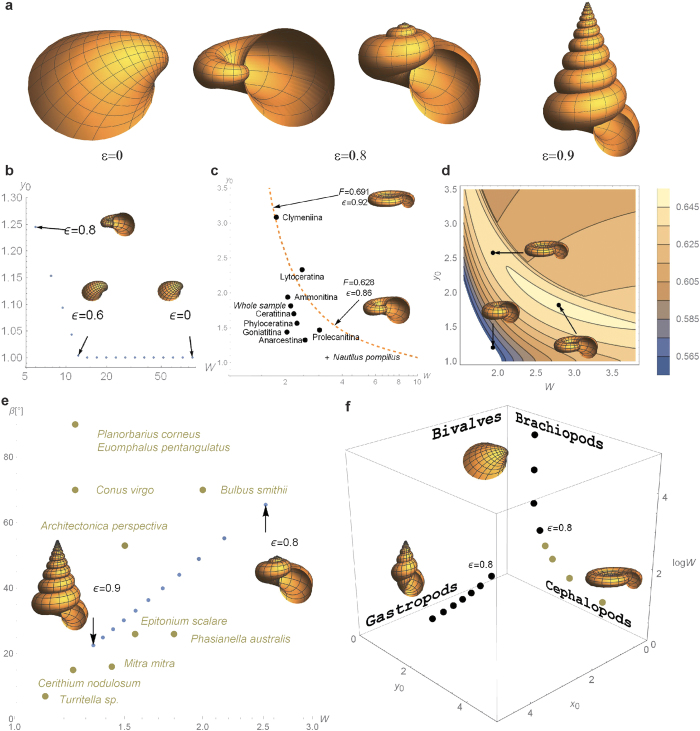
Optimal shape depends on the thickness exponent *ε.* (**a**) Optimal shapes resemble three basic shapes of natural shells, namely the bivalve shell, the flat-coiled shell, and the conical-coiled shell. (Left) *ε* = 0: (*x*_0_, *y*_0_, log*W*) = (0, 1, 4.45) (*F* = 0.175). (Left center) *ε* = 0.8: (*F* = 0.580). (Right center) *ε* = 0.8: (*x*_0_, *y*_0_, log*W*) = (1.61, 1.12, 0.93) (*F* = 0.580). (Right) *ε* = 0.9: (*x*_0_, *y*_0_, log*W*) = (5.67, 1.27, 0.29) (*F* = 0.683). (**b**) Optimal values of *y*_0_ and *W (x*_0_ = 0). (**c**) A dashed curve is the locus of *y*_0_ and *W* that maximize *F* under the constraint of *x*_0_ = 0. Ammonoid suborders are indicated according to Raup’s biometric data^19^. (**d**) A contour plot of *F* in the *W-y*_0_ plane (*ε* = 0.88). (**e**) The semi-angle *β* at the apex of optimal shapes is plotted against *W (ε* ≥ 0.8). Biometric data are due to Thompson^1^. (**f**) Optimal values of (*x*_0_, *y*_0_, log*W*) are plotted in a three-dimensional morphospace. According to Raup^10^, the regions in which representatives of four major taxonomic groups are concentrated are indicated by letters.
