# Supplementary material for: Optimal designs of mollusk shells from bivalves to snails
Source: Sci Rep. 2017 Feb 10;7:42445. doi: 10.1038/srep42445 (PMC5301254; doi:10.1038/srep42445)
Supplement: Supplementary Information [file srep42445-s1.pdf]

Supplementary Information for

Optimal designs of mollusk shells from bivalves to snails

Takuya Okabe (okabe.takuya@shizuoka.ac.jp), Jin Yoshimura (yoshimura.jin@shizuoka.ac.jp)

Materials and Methods
Figures S1-S7
References

Supplementary Informations:

Materials and Methods:

Outline

To illustrate the method, consider a right circular cone for a limpet. The cone surface is generated from a circular aperture of radius 1 centered at (x_0, y_0) (Fig. S1). For the moment, let us investigate a right cone for $y_0 = 0$. When the radius of the base circle is T, height of the cone is Tx_0. Independently of size T, the ratio of height to radius of the cone is x_0. The problem is to find an optimal value of x_0. The volume enclosed by the cone is the height $x_0 T$ times the base area πT^2 divided by 3, i.e., $V = \pi x_0 T^3/3$. The surface area of the right circular cone is given by π times radius T times slant length $\sqrt{(x_0 T)^2 + T^2}$, i.e., $S = \pi\sqrt{x_0^2 + 1}T^2$. Since thickness is not uniform, the volume of shell material V_s and the surface area S are related through a differential equation. The thickness h at the base of radius T is related to the initial thickness h_1 at radius 1 ($T = 1$) by $h = h_1 T^{\varepsilon}$, where ε is the scaling exponent of thickness. For a vertical slice between height T and $T + dT$, the volume of shell materials dV_s and the surface area dS are related by $dV_s = h(T)dS$. Therefore, $V_s \propto \sqrt{x_0^2 + 1}h_1 T^{2+\varepsilon}$. By eliminating size T, the inner volume V and the shell volume V_s satisfy a scaling relation $V = F(V_s/h_1)^{3/(2+\varepsilon)}$. The factor F defined by this equation takes a maximum at $x_0 = \sqrt{(2+\varepsilon)/(1-\varepsilon)}$, because $F \propto x_0(x_0^2 + 1)^{-3/(4+2\varepsilon)}$. This result is consistent with the main results. Optimal shape has a non-trivial ε-dependence in the limit of isometry ($\varepsilon = 1$). The optimal ratio of height to radius (x_0) is $\sqrt{2}$ for $\varepsilon = 0$. This is substantially higher than a typical ratio 0.68 of real limpets and even higher than an optimal ratio 1.06 to minimize lift and drag forces in turbulent flow.[1]

A coiled shell is analyzed in a similar manner. Also in this case, inner volume V, surface area S, thickness h and shell volume V_s vary as $V \propto w^3$, $S \propto w^2$, $h \propto w^{\varepsilon}$ and

$V_s \propto w^{2+\varepsilon}$, where w is a linear dimension of the shell aperture. Technical details are

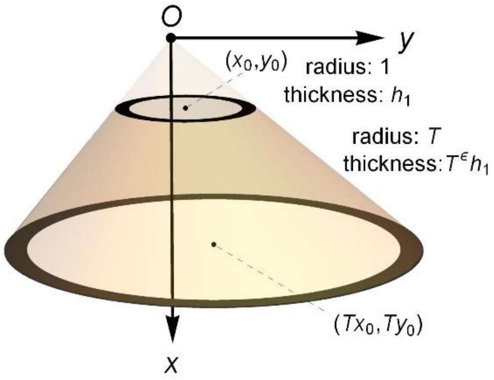

explained below.

Fig. S1. A circular cone for an uncoiled shell. The shell form is characterized by a scaling exponent of thickness (ε) and center coordinates (x_0, y_0) of an initial aperture of radius 1 and thickness h_1. The interest of the present study lies in the shape (x_0, y_0) dependence, not in the size (T) dependence. The enclosed volume $(\propto T^3)$ is independent of obliqueness (y_0), while the surface area $(\propto T^2)$ is minimized for a right cone ($y_0 = 0$). Sharpness of optimal shape (x_0) depends on the thickness exponent (ε).

Thickness Exponent

In practice, it is not easy to evaluate the scaling exponent ε based on the power-law relation $V \propto V_s^{3/(2+\varepsilon)}$ between inner volume V and shell volume V_s, because ε-dependence is not strong. Indeed, the proportional relation $V \propto V_s$ (shell weight is proportional to shell volume) has been taken for granted as a matter of course.[2] The actual value of the thickness exponent (ε) appears to have not been investigated. Here we attempted to estimate it based on Trueman's data for actual specimens of fossil ammonites and obtained $\varepsilon \simeq 0.8$ by the least-squares power-law fit (Fig. S2).[3] This result is consistent with Trueman's observation that "the volume of a shell increases, not in the ratio of the diameter^3 but rather in the relation to the $\text{diameter}^{2.7-2.8}$" (p.343, ref.3). As noted above and explained below, the shell material increases as $V_s \propto w^{2+\varepsilon}$, where w is a length of the shell.

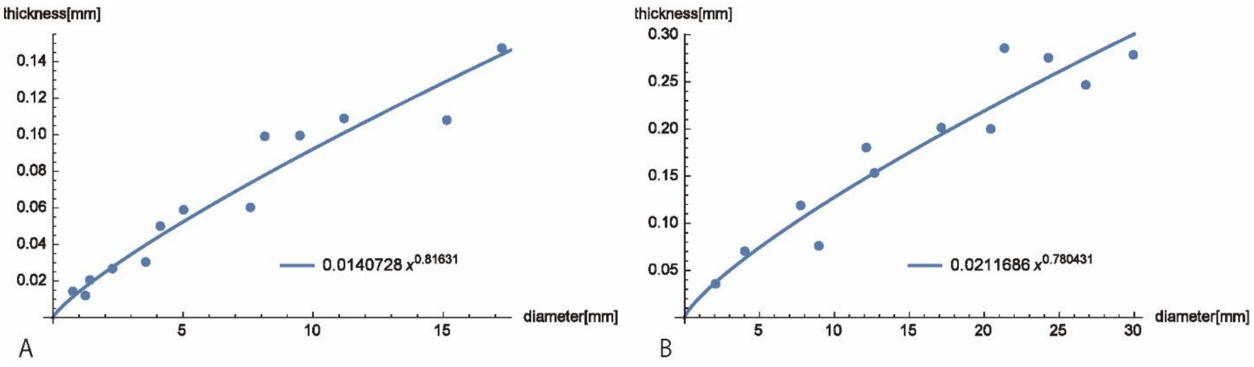

Fig. S2. Average thicknesses of ammonite shell at different diameters (ref.3), The least-squares fit by Ax^{ε} is drawn with a solid line. (**A**) *Promicroceras marstonense.* (**B**) *Dactylioceras commune.*

Technical Details

We use basic formulas of vector analysis[4], which was established decades after the time of Moseley[5,6], the pioneer of mathematical shell morphology. Today vector analysis is a necessary skill of a physicist, but surely not of a biologist. Readers may skip to Summary and Remarks. In short, what follows is a derivation and generalization of mathematical expressions used in prior studies[2,5-12].

Formulas of differential geometry

The position vector $(\vec{r})$ and the Cartesian coordinates (x, y, z) of a point on a surface are given as functions of two independent parameters s and t, i.e.,

$$\vec{r}(s,t) = (x(s,t), y(s,t), z(s,t)). \tag{1}$$

The surface area is given by

$$S = \iint \left| \frac{\partial \vec{r}}{\partial s} \times \frac{\partial \vec{r}}{\partial t} \right| dsdt. \tag{2}$$

This is as an integral of the magnitude (vertical bars) of the cross product $(\times)$ of two partial derivatives of the position vector $(\vec{r}(s,t))$. This formula is found by searching with the keywords "the surface area of a parametric surface".

According to "the divergence theorem", the volume bounded by the surface is given by

$$V = \iiint \operatorname{div} \vec{p}(\vec{r}) dV = \iint \vec{p}(\vec{r}) \cdot \left(\frac{\partial \vec{r}}{\partial s} \times \frac{\partial \vec{r}}{\partial t} \right) dsdt\,, \tag{3}$$

where $\vec{p}(\vec{r}) = (p_x(\vec{r}), p_y(\vec{r}), p_z(\vec{r}))$ is a vector field satisfying

$$\operatorname{div} \vec{p}(\vec{r}) = \frac{\partial p_x}{\partial x} + \frac{\partial p_y}{\partial y} + \frac{\partial p_z}{\partial z} = 1. \tag{4}$$

For $\vec{p}(\vec{r}) = (0,0,z)$,

$$V = \iint z \left(\frac{\partial x}{\partial s}\frac{\partial y}{\partial t} - \frac{\partial y}{\partial s}\frac{\partial x}{\partial t} \right) dsdt\,. \tag{5}$$

For $\vec{p}(\vec{r}) = (x/2, y/2, 0)$,

$$\frac{dV}{dt} = \frac{1}{2} \int \left(x \left(\frac{\partial y}{\partial s}\frac{\partial z}{\partial t} - \frac{\partial z}{\partial s}\frac{\partial y}{\partial t} \right) + y \left(\frac{\partial z}{\partial s}\frac{\partial x}{\partial t} - \frac{\partial x}{\partial s}\frac{\partial z}{\partial t} \right) \right) ds\,. \tag{6}$$

These formulas are so basic that it is difficult to cite the original source.

Uncoiled shells

A circular cone in Fig. S1 is represented by the parametric surface of

$$\vec{r}(s,t) = t(x_0, y_0 + \cos s\,, \sin s), \tag{7}$$

where two parameters t and s represent height $(0 < t < T)$ and arc length (or angle in radians) around the apertural circle of unit radius $(0 < s < 2\pi)$, respectively.

Substituting Eq. (7) into Eq. (2), the surface area is given by

$$S(T) = \int_0^T t\,dt \int_0^{2\pi} \sqrt{x_0^2 + (y_0 \cos s + 1)^2}\, ds. \tag{8}$$

Therefore, the T-derivative of $S(T)$ is given by

$$S'(T) = TS'(1), \tag{9}$$

where

$$S'(1) = \int_0^{2\pi} \sqrt{x_0^2 + (y_0 \cos s + 1)^2}\, ds. \tag{10}$$

This integral takes a minimum value at $y_0 = 0$, i.e., a right circular cone. Indeed, by expanding $S'(1)$ around $y_0 = 0$,

$$S'(1) = 2\pi\sqrt{x_0^2 + 1} + \frac{1}{2}\frac{\pi x_0^2}{(x_0^2 + 1)^{3/2}} y_0^2 + \cdots. \tag{11}$$

Thus, the surface area $S(T) = T^2 S'(1)/2$ takes a minimum value $\pi\sqrt{x_0^2 + 1}T^2$ at $y_0 = 0$. This result is obtained by an elementary formula (see Outline).

The volume of shell materials dV_s and the surface area dS are related by

$$dV_s = h(T)dS, \tag{12}$$

where $h(T)$ is the thickness at the height T. As shown in Fig. S1, the scaling exponent ε of thickness is introduced through the size T dendence of thickness $h(T)$,

$$h(T) = h_1 T^{\varepsilon}, \tag{13}$$

where h_1 is thickness for $T = 1$. Note that T for the parameter t represents the height of the cone and the radius of the base circle. By Eqs. (9), (12) and (13), the shell volume V_s is given by

$$V_s = \frac{S'(1)h_1}{2+\varepsilon} T^{2+\varepsilon}. \tag{14}$$

Substituting Eq. (7) into Eq. (5), the cone volume is given by

$$V(T) = x_0 \int_0^T t^2\, dt \int_0^{2\pi} (\sin s)^2 ds = \frac{\pi}{3} x_0 T^3, \tag{15}$$

which is independent of y_0. This result is obtained by an elementary formula (see Outline).

To summarize,

$$V = F(V_s/h_1)^{3/(2+\varepsilon)}, \tag{16}$$

where

$$F = \frac{\pi}{3} x_0 \left(\frac{2+\varepsilon}{S'(1)}\right)^{3/(2+\varepsilon)}. \tag{17}$$

This factor F takes a maximum at

$$(x_0, y_0) = \left(\sqrt{\frac{2+\varepsilon}{1-\varepsilon}}, 0\right). \quad (18)$$

Coiled shells

The parametric surface of a coiled shell is given by

$$\vec{r}(s,\theta) = e^{\theta\cot\alpha} R_x(\theta)\vec{r}_a(s). \quad (19)$$

An initial aperture at angle $\theta = 0$ is represented by $\vec{r}_a(s)$ (Fig. S3). The parameter θ is the angle of rotation about the shell axis (the x-axis). The rotation by angle θ about the x-axis is represented by $R_x(\theta)$. In the matrix representation, it is given by

$$R_x(\theta) = \begin{pmatrix} 1 & 0 & 0 \\ 0 & \cos\theta & -\sin\theta \\ 0 & \sin\theta & \cos\theta \end{pmatrix}. \quad (20)$$

The aperture lying in the plane of the rotation axis (the xy plane) is parametrically represented as

$$\vec{r}_a(s) = (x_a(s), y_a(s), 0). \quad (21)$$

This equation defines the x and y-coordinates of the initial aperture (x_a and y_a) as functions of arc length s. This is shown as C in Fig. S3. In general cases, the apertural curve $\vec{r}_a(s)$ may also have a z-component. The aperture used in the main text (Fig. 1a) is an unit circle centered at (x_0, y_0), namely

$$x_a(s) = x_0 + \cos s, y_a(s) = y_0 + \sin s. \quad (22)$$

The parameter s varies from 0 to 2π. Rotation by angle θ about the x-axis brings the position vector of Eq. (21) to

$$R_x(\theta)\vec{r}_a(s) = (x_a(s), y_a(s)\cos\theta, y_a(s)\sin\theta). \quad (23)$$

For $0 < s < 2\pi$, this is shown as C_1 in Fig. S3. The multiplication factor $e^{\theta\cot\alpha}$ in Eq. (19) represents uniform expansion from the origin O (shell apex). This is the scale factor w noted above in Outline,

$$w = e^{\theta\cot\alpha}. \quad (24)$$

For a full turn of $\theta = 2\pi$, this factor is the expansion ratio between successive whorls,

$$W = e^{2\pi\cot\alpha}. \quad (25)$$

Hence, in an explicit expression,

$$\vec{r}(s,\theta) = (e^{\theta\cot\alpha}x_a(s), e^{\theta\cot\alpha}y_a(s)\cos\theta, e^{\theta\cot\alpha}y_a(s)\sin\theta). \quad (26)$$

For $0 < s < 2\pi$, this is shown as C_2 in Fig. S3. The cross-sections of the surface in the initial plane ($z = 0$, $y > 0$) are obtained by substituting $\theta = 0, 2\pi, 4\pi, \cdots$, i.e.,

$$\vec{r}(s,0) = \vec{r}_a(s), \vec{r}(s,2\pi) = W\vec{r}_a(s), \vec{r}(s,4\pi) = W^2\vec{r}_a(s), \cdots. \quad (27)$$

In terms of the scale factor of Eq. (24), thickness of the aperture at angle θ is given by

$$h(\theta) = w^\varepsilon h_0 = (e^{\theta\cot\alpha})^\varepsilon h_0, \quad (28)$$

where a scaling exponent (ε) and thickness at $\theta = 0$ ($h_0 = h(0)$) are introduced. It is assumed that thickness is independent of the parameter s, i.e., thickness is uniform around the aperture. As in Eq. (21), this assumption is made just for simplicity.
Cross-sectional thicknesses in the plane of $\theta = 0$ are given by

$$h_0, W^{\varepsilon} h_0, W^{2\varepsilon} h_0, \cdots. \quad (29)$$

These are the mathematical representation of shell form graphically illustrated in Fig. 1a.

The spiral seen in a plane perpendicular to the coiling axis is called the equiangular spiral because the angle made between the tangent and radius of the spiral is constant independently of the distance from the center[2]. The scale factor of Eq. (24) is expressed in terms of the cotangent of the angle α of this equiangular spiral. Hence, $W > 1$ for a practical range of $0 < \alpha < \pi/2$. In an unrealistic limit of $\alpha = \pi/2$, $W = 1$, for which the spiral reduces to a circle.

To express the total volume V in terms of the shape parameters (x_0, y_0, W), it is instructive to note that the volume V increases in proportion to the third power of size (w^3) for a sufficiently large shell $(w \gg 1)$. If the volume V is regarded as a function of θ, the volume $V(\theta)$ as well as its its θ-derivative $\dot{V}(\theta)$ are proportional to $w^3 = e^{3\theta \cot\alpha}$. The θ-derivative $\dot{V}(\theta)$ is given by Eq. (6) for $t = \theta$. Therefore, the internal volume is given by

$$V(\theta) = \int_0^{\theta} \dot{V}(\theta) d\theta = \frac{\dot{V}(0)}{3\cot\alpha}\left(e^{3\theta\cot\alpha} - 1\right). \quad (30)$$

Substituting Eqs. (19) and (21) into Eq. (6) for $t = \theta$,

$$\dot{V}(0) = \frac{1}{2}\int y_a (x_a y_a{}' - y_a x_a{}') ds. \quad (31)$$

The prime symbol means the derivative with respect to s. In a similar manner, the surface area $S(\theta)$ is related to its θ-derivative by

$$S(\theta) = \int_0^{\theta} \dot{S}(\theta) d\theta = \frac{\dot{S}(0)}{2\cot\alpha}\left(e^{2\theta\cot\alpha} - 1\right). \quad (32)$$

Note that $S \propto w^2 = e^{2\theta\cot\alpha}$. Substituting Eqs. (19) and (21) into Eq. (2) for $t = \theta$,

$$\dot{S}(0) = \int \sqrt{y_a^2 (x_a{}'^2 + y_a{}'^2) + (x_a y_a{}' - x_a{}' y_a)^2 (\cot\alpha)^2} ds. \quad (33)$$

As in Eq. (12), the shell material volume $V_s(\theta)$, the surface area $S(\theta)$ and the thickness $h(\theta)$ are related by

$$\frac{dV_s}{d\theta} = h(\theta)\frac{dS}{d\theta}. \quad (34)$$

Therefore,

$$V_s(\theta) = \int_{-\infty}^{\theta} h(\theta)\dot{S}(\theta)d\theta = \frac{h_0\dot{S}(0)}{(2+\varepsilon)\cot\alpha}\left(e^{(2+\varepsilon)\theta\cot\alpha} - 1\right). \quad (35)$$

For a sufficiently large shell ($w \gg 1$), V in Eq. (30) and V_s in Eq. (35) are proportional to $w^3 = e^{3\theta\cot\alpha}$ and $w^{2+\varepsilon}$, respectively. A scaling relation between V and V_s is obtained by eliminating w from these expressions.

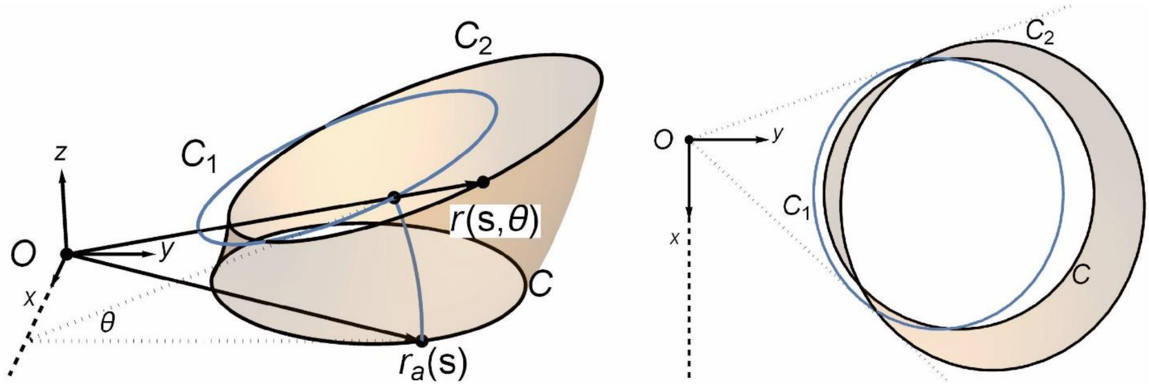

Fig. S3. Construction of a coiled shell surface. C is an aperture in the xy plane. A point on C is represented by a position vector $\vec{r}_a(s)$ as a function of a parameter s $(0 < s < 2\pi)$. C_1 is obtained by rotating C by angle θ about the coiling axis (the x-axis). A new aperture C_2 is obtained from C_1 by expanding C_1 by a constant factor ($w = e^{\theta\cot\alpha} > 1$), where the coordinate origin O is the center of expansion. The radius of C_2 is w times as large as that of C and C_1. The position vector $\vec{r}(s,\theta)$ of a point on C_2 is related to $\vec{r}_a(s)$ on C by Eq. (19). By definition, $\vec{r}(s,0) = \vec{r}_a(s)$. Thus, the curved surface is parametrically represented by the position vector $\vec{r}(s,\theta)$, where parameters s and θ are coordinates on the surface. Note that the resulting shell surface depends not only on the expansion factor w but also on the center coordinates (x_0, y_0) of the initial aperture C. If the center lies on the y-axis ($x_0 = 0$) and the expantion rate w is close to 1, the shell surface resembles an ammonite form.

Summary

The internal volume V and shell material volume V_s of a coiled shell satisfy a scaling relation

$$V = F(V_s/h_0)^{3/(2+\varepsilon)}, \tag{36}$$

where h_0 is thickness of the initial aperture of radius 1, ε is the scaling exponent of thickness (Fig. 1a), and

$$F = \frac{1}{3}(2+\varepsilon)^{\frac{3}{2+\varepsilon}}(\cot\alpha)^{\frac{1-\varepsilon}{2+\varepsilon}}\frac{\dot{V}(0)}{\dot{S}(0)^{3/(2+\varepsilon)}}. \tag{37}$$

By Eq. (25),

$$\cot\alpha = \frac{\log W}{2\pi}, \tag{38}$$

where W is the whorl expansion ratio. The factor F in Eq. (37) is calculated as a function of x_0, y_0 and W by substituting Eq. (22) into Eqs. (31) and (33) for $\dot{V}(0)$ and $\dot{S}(0)$, which are differential volume and surface area of the shell. As noted in the main text, the factor $(\cot\alpha)^{\frac{1-\varepsilon}{2+\varepsilon}}$ in Eq. (37) indicates that F is not well-behaved in the limit of $\varepsilon \to 1$ and $W \to 1$ $(\cot\alpha \to 0)$, where zero $(\cot\alpha)$ is raised to the power zero $(1-\varepsilon)$. In order for Eqs. (31) and (33) to be used in Eqs. (30) and (35), respectively, it should be taken into account that the aperture is not fully deposited by shell material when the shell surface overlaps with the rotation axis and/or the past whorl (see below).

Remarks

Equations (30) and (31) are equivalent to Moseley's volume formula[3]. The surface-area formula of Eqs. (32) and (33) agrees with Moseley's initial result only in the limit of $W = 1$ $(\cot\alpha = 0)$[5,11] and is reducible to an expression equivalent to Moseley's corrected result[6,12]. Since thickness is not uniform in general, the surface area in Eq. (32) has no biological significance for the present purpose.

The apertural area, i.e., the area inside the apertural curve of Eq. (21), is given by

$$A = \frac{1}{2}\int (x_a y_a' - y_a x_a')ds. \tag{39}$$

(Green's theorem.) Therefore, Eq. (31) may be written as

$$\dot{V}(0) = R_a A, \tag{40}$$

where R_a represents the distance from the coiling axis of a center of the aperture (the y-coordinate of the center of mass of the aperture). Under the assumption of isometric growth $(\varepsilon = 1)$, Raup evaluated both internal shell volume and volume of shell

material by using the inner volume between angle $-\theta$ up to the aperture at $\theta = 0$,

$$\int_{-\theta}^{0} \dot{V}(\theta) d\theta = \frac{1 - e^{-3\theta\cot\alpha}}{3\cot\alpha} \dot{V}(0), \tag{41}$$

along with Eq. (40). Moreover, he assumed $h_0 = 0.0772$ for isometric thickness[7,10]. Similarly, the perimeter of the aperture is given by

$$P = \int \sqrt{x_a'^2 + y_a'^2} ds. \tag{42}$$

In the limit $W = 1$ ($\cot\alpha = 0$), Eq. (33) reduces to

$$\dot{S}(0) = R_p P, \tag{43}$$

where R_p is the distance from the coiling axis of a center of the apertural perimeter (the y-coordinate of the center of mass of the perimeter). For isometric shells ($\varepsilon = 1$), the ratio of inner volume to shell volume is proportional to the ratio of the area to perimeter of the aperture. According to Eqs. (36) and (37),

$$\frac{V}{V_s} = \frac{F(\varepsilon = 1)}{h_0} = \frac{\dot{V}(0)}{h_0 \dot{S}(0)} = \frac{R_a A}{h_0 R_p P} \simeq \frac{A}{h_0 P}. \tag{44}$$

This ratio is independent of the scale factor w ($A \propto w^2$, $P \propto w$, and $h_0 \propto w$ by the assumption of isometry). Therefore, the ratio may be estimated from an aperture at an arbitrary instant (at any angle θ). The expression of Eq. (44) has been used as a measure of shell material efficiency[8,9]. Thus, the scaling relation of the present study (Eq. (36)) is a natural generalization of the previous results for the isometric case ($\varepsilon = 1$).

The symbol α is occasionally used with different meanings[2,11]. In the present study, α is defined by the scale factor in Eq. (24), or in Eq. (25). Accordingly, the angle α is constant for a given value of W, namely

$$\alpha = \cot^{-1} \frac{\log W}{2\pi}. \tag{45}$$

In particular, this angle does not depend on the apex angle of a turbinate shell. As noted above, α is the angle of the equiangular spiral in a plane perpendicular to the coiling axis. In contrast, in his second paper, Moseley regarded a three-dimensional equiangular spiral on a turbinate shell as a two-dimensional (plane) equiangular spiral wrapped upon an imaginary cone surface[6]. Then, the whorl expansion ratio is given by $W = e^{2\pi\cot A \sin i}$ where i is half the angle at the apex of the imaginary cone and A is the angle of the plane equiangular spiral. Therefore,

$$\cot\alpha = \cot A \sin i. \tag{46}$$

In the first paper, Moseley used the same symbol A for α (ref.5). Thompson did not distinguish i and the semi-angle at the apex angle of a turbinate shell, β (ref.2).

Referring to Moseley's second paper, Raup and Graus wrongly replaced $\cot\alpha$ (or $\cot A$) in the first paper of Moseley by $\sqrt{1+T^2}\log W/(2\pi)$ because $\sin i \simeq \sin\beta = 1/\sqrt{1+T^2}$ in terms of Raup's translation rate T (ref.11). The last equation is also incorrect (see Eq. (60)). Moseley's i is defined for each point on the aperture. In the notation of Eq. (21),

$$i = \tan^{-1}\frac{y_a(s)}{x_a(s)}. \tag{47}$$

In the main text, we used mean values in Table 1 of Raup[7] for Fig. 2d and a table in page 818 of Thompson[2] for Fig. 3d.

Non-overlapping whorls

There is no problem when the aperture is fully deposited by shell material. To evaluate $\dot{V}(0)$ and $\dot{S}(0)$ for F in Eq. (37), the parameter s may be set to vary in a single period from 0 to 2π, i.e.,

$$\dot{V}(0) = V(0,2\pi) \tag{48}$$

and

$$\dot{S}(0) = S(0,2\pi), \tag{49}$$

where

$$V(s_-, s_+) = \frac{1}{2}\int_{s_-}^{s_+} y_a\,(x_a y_a{}' - y_a x_a{}')ds \tag{50}$$

and

$$S(s_-, s_+) = \int_{s_-}^{s_+} \sqrt{y_a^2(x_a{}'^2 + y_a{}'^2) + (x_a y_a{}' - x_a{}' y_a)^2(\cot\alpha)^2}\,ds. \tag{51}$$

In these expressions, x_a and y_a in Eq. (22) are substituted for integration.

Effect of whorl overlap

Case I: $1 < r_0 < (W+1)/(W-1)$ and $y_0 > 1$ (Fig. S4)

If the revolving surface overlaps with a preceding whorl, the limits of integration in $\dot{V}(0)$ and $\dot{S}(0)$ have to be properly modified so as to exclude the overlapped portion (the aperture is not fully deposited). For the plane curve in the xy-plane (Eq. (21)), intersection points on successive whorls satisfy

$$\vec{r}_a(s_i) = W^{-1}\vec{r}_a(s_i'), \tag{52}$$

where $W^{-1}\vec{r}_a(s_i')$ represents a point on the previous whorl. This equation is geometrically solved by drawing the plane curves of two apertures $\vec{r}_a(s)$ and $W^{-1}\vec{r}_a(s)$ (Fig. S4). For this purpose, it is convenient to use W instead of $\cot\alpha$ (Eq. (25)). As shown in Fig. S4, successive whorls overlap if $r_0 > 1$ and

$$1 + W^{-1} > (1 - W^{-1})r_0, \tag{53}$$

or if

$$1 < r_0 < \frac{W+1}{W-1}, \tag{54}$$

where

$$r_0 = \sqrt{x_0^2 + y_0^2}. \tag{55}$$

is the distance between the center of the aperture $\vec{r}_a(s)$ and the expansion center O at the coordinate origin. If the whorls do not touch the rotation axis (Fig. S4), namely

$$y_0 > 1, \tag{56}$$

then

$$\dot{S}(0) = S(s_1, s_2), \tag{57}$$

and

$$\dot{V}(0) = V(s_1, s_2) - W^{-3}V(s_1', s_2'), \tag{58}$$

where

$$\begin{aligned} s_1 &= -\pi + \varphi + \psi, \\ s_2 &= \pi - \varphi + \psi, \\ s_1' &= -\varphi' + \psi, \\ s_2' &= \varphi' + \psi, \\ \psi &= \tan^{-1}(y_0/x_0), \\ \varphi &= \cos^{-1}\frac{W^2 + r_0^2(W-1)^2 - 1}{2r_0W(W-1)}, \\ \varphi' &= \cos^{-1}\frac{1 + r_0^2(W-1)^2 - W^2}{2r_0(W-1)}. \end{aligned} \tag{59}$$

Geometrical meanings of these symbols are shown in Fig. S4. In the right-hand side of Eq. (58), the first term $V(s_1, s_2)$ counts the volume bounded by the outer whorl in

Fig. S4 (solid line), while the second term $W^{-3}V(s_1', s_2')$ subtracts the volume inside the inner whorl (dashed line).

The semi-angle of the cone apex β is given by

$$\beta = \beta' + \psi = \sin^{-1}\frac{1}{r_0} + \tan^{-1}\frac{y_0}{x_0}, \quad (60)$$

which is used in Fig. 3d of the main text.

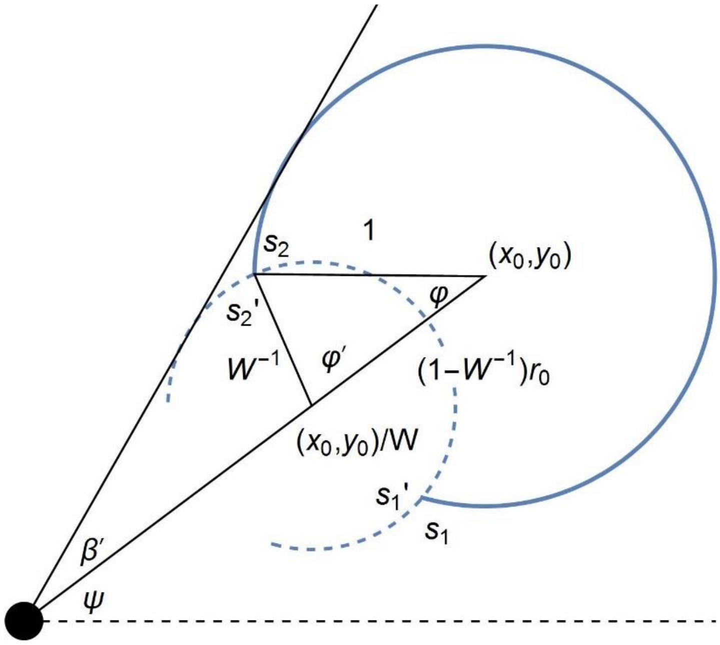

Fig. S4. Case I. $1 < r_0 < (W+1)/(W-1)$ and $y_0 > 1$. A solid circle of radius 1 represents $\vec{r}_a(s)$, while a dashed circle of radius W^{-1} represents $\vec{r}_a(s)/W$. The two circles cross at two points satisfying $\vec{r}_a(s_1) = \vec{r}_a(s_1')/W$ and $\vec{r}_a(s_2) = \vec{r}_a(s_2')/W$. The distance from the origin (dot) to the center (x_0, y_0) of the former circle is denoted as r_0.

Case II: $1 < r_0 < (W+1)/(W-1)$, $0 < y_0 < 1$ and $s_1 < s_0'$ (Fig. S5)
This and following cases are not covered by Raup's model. If

$$0 < y_0 < 1, \tag{61}$$

the apertural curve crosses the axis (Fig. S5). The crossed portion ($s_0' < s < s_0$, $y_a(s) < 0$) should be properly taken into account in evaluating V and V_s .

$$\dot{S}(0) = S(s_1, s_0') + S(s_0, s_2) \tag{62}$$

and

$$\dot{V}(0) = V(s_1, s_0') + V(s_0, s_2) - W^{-3}V(s_1', s_2'), \tag{63}$$

where

$$\begin{aligned} s_0 &= -\psi', \\ s_0' &= -\pi + \psi', \end{aligned} \tag{64}$$

and

$$\psi' = \sin^{-1} y_0. \tag{65}$$

The same results are simply obtained by requiring $y_a(s) > 0$ in the numerical integration of $\dot{S}(0)$ and $\dot{V}(0)$.

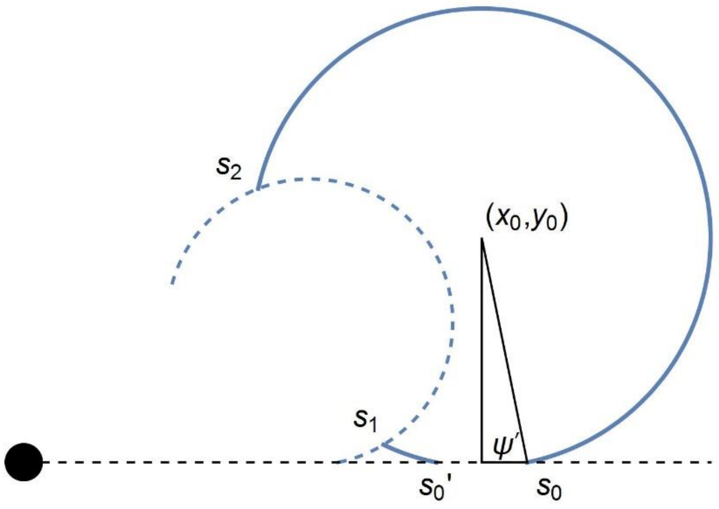

Fig. S5. Case II. $0 < y_0 < 1$ and $s_1 < s_0'$.

Case III: $\mathbf{1} < \boldsymbol{r_0} < (W+1)/(W-1)$**,** $\mathbf{0} < \boldsymbol{y_0} < 1$ **and** $\boldsymbol{s_0}' < \boldsymbol{s_1}$ **(Fig. S6)**

In this case, there is no hollow space around the axis (Fig. S6). The lower limit of integration is given by s_0 in Eq. (64).

$$\dot{S}(0) = S(s_0, s_2). \tag{66}$$

$$\dot{V}(0) = V(s_0, s_2) - W^{-3}V(s_0, s_2'). \tag{67}$$

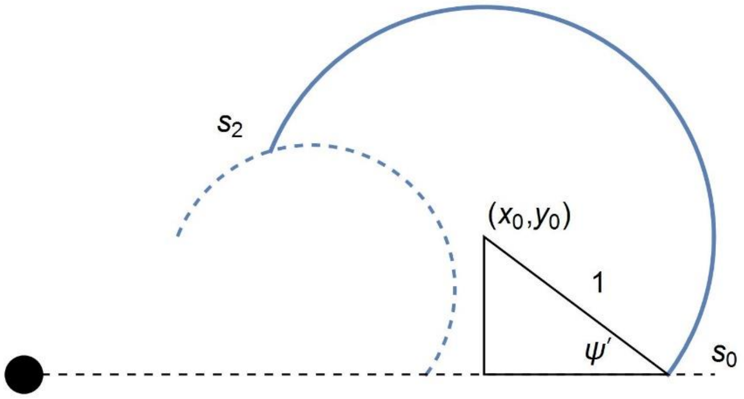

Fig. S6. Case III. $0 < y_0 < 1$ and $s_1 > s_0'$.

Case IV: $\mathbf{0} < \boldsymbol{r_0} < 1$ (Fig. S7)

Whorls do not touch each other (Fig. S7). The limits of integration are given by solutions $s = s_0$ and $s = \pi - s_0$ of $y_a(s) = 0$.

$$\dot{S}(0) = S(s_0, \pi - s_0). \tag{68}$$

$$\dot{V}(0) = (1 - W^{-3})V(s_0, \pi - s_0). \tag{69}$$

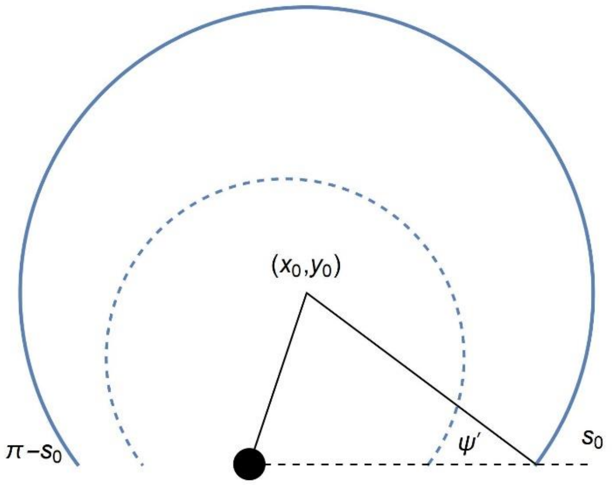

Fig. S7. Case IV. $0 < r_0 < 1$.

References:

1. Denny, M. Limits to optimization: fluid dynamics, adhesive strength and the evolution of shape in limpet shells. *Journal of Experimental Biology* **203**, 2603‑2622 (2000).
2. Thompson, D.W. *On Growth and Form*. (Cambridge University Press, 1942).
3. Trueman, A.E. The ammonite body-chamber, with special reference to the buoyancy and mode of life of the living ammonite. *Quarterly Journal of the Geological Society* **96**, 339‑383 (1940).
4. Colley, J.C., Leon, S. *Math 311 Linear Algebra and Vector Calculus (At Texas A&M)* (Pearson College Division, 2013).
5. Moseley, H. On the geometrical forms of turbinated and discoid shells. *Philosophical Transactions of the Royal Society of London* **128**, 351‑370 (1838).
6. Moseley, H. On chonchyliometry. *Philosophical Magazine* **21**, 300‑305 (1842).
7. Raup, D.M. Geometric analysis of shell coiling: Coiling in ammonoids. *Journal of Paleontology* **41**, 43‑65 (1967).
8. Heath, D.J. Whorl overlap and the economical construction of the gastropod shell. *Biological Journal of the Linnean Society* **24**, 165‑174 (1985).
9. Tendler, A. Mayo, A., Alon, U. Evolutionary tradeoffs, pareto optimality and the morphology of ammonite shells. *BMC Systems Biology* **9**, 12 (2015).
10. Raup, D.M. & Chamberlain, J.A. Equations for volume and center of gravity in ammonoid shells. *Journal of Paleontology* **41**, 566‑574 (1967).
11. Raup, D.M. & Graus, R.R. General equations for volume and surface area of a logarithmically coiled shell. *Journal of the International Association for Mathematical Geology* **4**, 307‑316 (1972).
12. Hutchinson, J.M.C. Three into two doesn’t go: twodimensional models of bird eggs, snail shells and plant roots. *Biological Journal of the Linnean Society* **70**, 161‑187 (2000).
